# Surviving within the amoebal exocyst: the *Mycobacterium avium *complex paradigm

**DOI:** 10.1186/1471-2180-10-99

**Published:** 2010-04-01

**Authors:** Iskandar Ben Salah, Michel Drancourt

**Affiliations:** 1 Unité de Recherche sur les Maladies Infectieuses et Tropicales Emergentes, UMR CNRS-6236, IRD 189, IFR 48 Faculté de Médecine, Université de la Méditerranée, Marseille France; 2 Assistance Publique des Hôpitaux de Marseille, Fédération de Microbiologie clinique Hôpital la Timone Marseille-France

## Abstract

**Background:**

Most of environmental mycobacteria have been previously demonstrated to resist free-living amoeba with subsequent increased virulence and resistance to antibiotics and biocides. The *Mycobacterium avium *complex (MAC) comprises of environmental organisms that inhabit a wide variety of ecological niches and exhibit a significant degree of genetic variability. We herein studied the intra-ameobal location of all members of the MAC as model organisms for environmental mycobacteria.

**Results:**

Type strains for *M. avium*, *Mycobacterium intracellulare*, *Mycobacterium chimaera*, *Mycobacterium colombiense*, *Mycobacterium arosiense*, *Mycobacterium marseillense*, *Mycobacterium timonense *and *Mycobacterium bouchedurhonense *were co-cultivated with the free-living amoeba *Acanthamoeba polyphaga *strain Linc-AP1. Microscopic analyses demonstrated the engulfment and replication of mycobacteria into vacuoles of *A. polyphaga *trophozoites. Mycobacteria were further entrapped within amoebal cysts, and survived encystment as demonstrated by subculturing. Electron microscopy observations show that, three days after entrapment into *A. polyphaga *cysts, all MAC members typically resided within the exocyst.

**Conclusions:**

Combined with published data, these observations indicate that mycobacteria are unique among amoeba-resistant bacteria, in residing within the exocyst.

## Background

So-called amoeba-resistant bacteria are characterized by the ability to survive within free-living amoeba (FLA) trophozoites [1,2]. Some amoeba-resistant species have been further demonstrated to survive within the amoebal cyst which may act as a "Trojan horse" protecting the organisms from adverse environmental conditions [1]. The amoebal cyst is comprised of the nucleus and the cytoplasm embedded into three successive layers, *i.e. *the endocyst, the clear region and the outer exocyst. Despite the fact that specific location of amoeba-resistant bacteria into the amoebal cyst could modify the outcome of the organisms, precise location of intracystic organisms has not been systematically studied.

Most of environmental mycobacteria have been demonstrated to be amoeba-resistant organisms also residing into the amoebal cyst [3] (Table 1). The *Mycobacterium avium *complex (MAC) organisms have been used as model organisms for environmental mycobacteria, comprising of mycobacteria that are responsible for opportunistic infections and zoonoses [4-8]. *M. avium *and *Mycobacterium intracellulare *have been recovered from various sources, including fresh water [9-13] and hospital water supplies, in which FLA are frequently isolated [14-17]. Several experimental studies have further demonstrated *M. avium*-FLA interactions, including *Acanthamoeba *spp. [3,18-22] and *Dictyostelium *spp. [23-25]. *M. avium *and *M. intracellulare *have also been grown in the ciliated, unicellular protist *Tetrahymena pyriformis *[26]. It has been demonstrated that *M. avium *subsp. *avium *and *M. avium *subsp. *paratuberculosis *are able to survive within FLA [20-22], which results in their increased virulence [18,19] and protection against adverse situations including exposure to antibiotics [19]. The habitat of the recently described *Mycobacterium chimaera *(formerly sequevar MAC-A), isolated from respiratory tract specimens [27-29]; *Mycobacterium colombiense *(formerly sequevar MAC-X), isolated from the blood of an HIV-positive patient [30] and from enlarged lymph nodes in non-immunocompromised children [30-32];*Mycobacterium arosiense *isolated from bone lesions [33]; and *Mycobacterium marseillense*, *Mycobacterium timonense *and *Mycobacterium bouchedurhonense *isolated from respiratory tract specimens [34,35], remains however unknown. MAC species exhibit on-going evolutionary divergence as evidenced by the 97.9-98.71% ANI (Average Nucleotide Identity) between the genomes of *M. avium *subsp. *paratuberculosis *K10 (NC_000962) and *M. avium *strain 104 (NC_008595), the 3.7% 16S rRNA gene divergence between *M. avium *and *M. timonense *and between *M. avium *and *M. chimaera*, and the 7.2% *rpo*B gene sequence divergence between *M. avium *and *M. colombiense *[34].

**Table 1:** Studies of interactions between MAC species and amoeba.

*Mycobacterium avium *Species	Strains	Amoeba species	Survival in *A. polyphaga*		Reference
				
			Trophozoites	Cysts	
*M. avium *subsp. *avium*	*M. avium *109	*A. castellanii*	+	?	[47]
*M. avium *subsp. *avium*	CIP104244^T^	*A. polyphaga *Linc-AP1	+	+	[3]
*M. intracellulare*	CIP104243^T^	*A. polyphaga *Linc-AP1	+	+	[3]
*M. avium *subsp.					
*paratuberculosis*	?	*A. castellanii *CCAP1501	+	?	[22]
*M. avium *subsp.					
*paratuberculosis*	?	*A. castellanii *CCAP1501	+	+	[20]
*M. avium *subsp. *avium*	?	*D. discodium *AX2	+	?	[24]
*M. avium *subsp. *avium*	?	*A. castellanii*	+	?	[48]
*M. avium *subsp. *hominissuis*	*M. avium *104	*A. castellanii *ATCC30234	+	?	[49]
*M. avium*	Serotype 4	*A. castellanii *ATCC30872	+	+	[21]
*M. avium*	?	*A. castellanii *ATCC30234	+	+	[18]
*M. avium *subsp. *avium*	ATCC 25291^T^	*A. polyphaga *Linc-AP1	+	+	Present study
*M. avium *subsp.					
*paratuberculosis*	ATCC 19698^T^	-	+	+	-
*M. avium *subsp. *hominissuis*	IWGMT 49	-	+	+	-
*M. avium *subsp. *silvaticum*	ATCC 49884^T^	-	+	+	-
*M. intracellulare*	ATCC 15985	-	+	+	-
*M. chimaera*	DSM 446232^T^	-	+	+	-
*M. colombiense*	CIP 108962^T^	-	+	+	-
*M. marseillense*	CSUR P30^T^	-	+	+	-
*M. timonense*	CSUR P32^T^	-	+	+	-
*M. bouchedurhonense*	CSUR P34^T^	-	+	+	-
*M. arosiense*	DSM 45069^T^	-	+	+	-

Using optic microscopy, electron microscopy and culturing methods, we herein used the MAC species as model organisms to study the location of environmental mycobacteria into the amoebal cyst and we further compared these observations with previously published data to find out that residing into the exocyst is a unique characteristic of environmental mycobacteria among amoeba-resistant organisms.

## Results and Discussion

**Figure 1:**
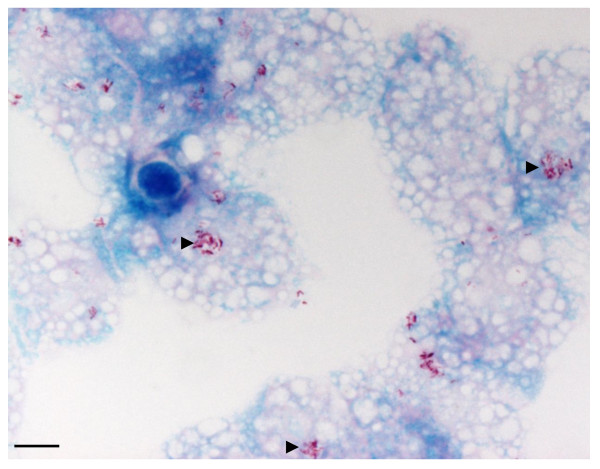
**Clusters of *Mycobacterium colombiense *(▶) in trophozoïtes of the free-living amoeba *Acanthamoeba polyphaga *Linc-AP1 (Ziehl Neelsen staining after a 24-hour incubation at 32°C)**. Scale bar = 10 μm.

**Figure 2:**
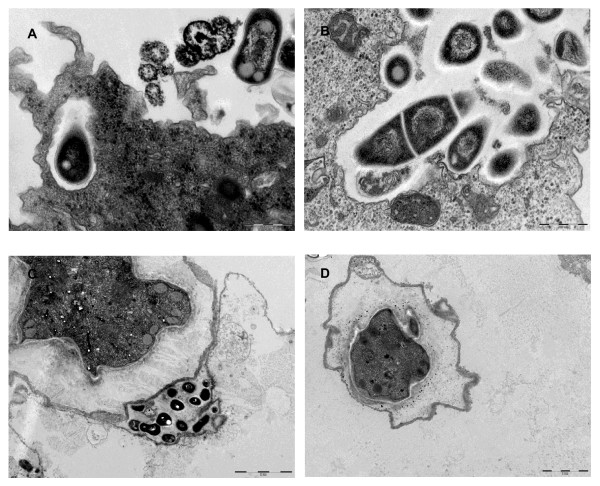
**Transmission electron-microscopy images of trophozoites and amoebal cysts infected by ***M. colombiense *(A and B. Scale bar = 500 nm), *M. avium, M. marseillense *(C, D and E. Scale bar = 2 μm) Ec: Exocyst, Ed: Endocyst, Cr: Clear region, M: *Mycobacterium*, P: Phagosome.

The 11 MAC strains (8 species) studied survived, but did not grow, after a 24-hour incubation in Page's modified Neff's Amoeba Saline (PAS) at 32°C. Microscopic examination of infected amoeba demonstrated that all MAC organisms were entrapped in *A. polyphaga *trophozoites and were visible in 3- to 5-μm large "*Mycobacterium *containing vacuoles" as early as 24 hours post-infection; 1 to 12 such vacuoles were observed per infected amoeba (Figure 1). The mean number of "*Mycobacterium *containing vacuoles" was not statistically different between the various MAC species. Electron microscopy observations revealed that, in the "*Mycobacterium *containing vacuoles" containing only one organism, there was a close apposition of the vacuole membrane all over the mycobacterial cell surface (Figure 2A, B), which was tightly apposed all over the organism cell wall, in contrast to organisms in vacuoles that contained several organisms as previously described in macrophages [36]. In this study, we did not resolved whether the presence of several mycobacteria within one vacuole resulted from the uptake of clumped mycobacteria, the replication of mycobacteria or the coalescence of several, single-organism vacuoles remains undetermined. In any case, our observations agree with previous studies that *M. avium *is initially entrapped in the vacuoles of amoebal trophozoites [18,23,24,21,22] and macrophages [36] (Table 1). In *Dictyostelium*, *M. avium *accumulated within vacuoles decorated with vacuolin, the *Dictyostelium *flotilin homologue, but it did not break the vacuole membrane, in contrast to *Mycobacterium tuberculosis *and *Mycobacterium marinum*. This result was linked to the absence of a particular region of difference (RD1), which in *M. tuberculosis *and *M. marinum*, encodes a type seven secretion system along with secreted effectors [23].

**Figure 3:**
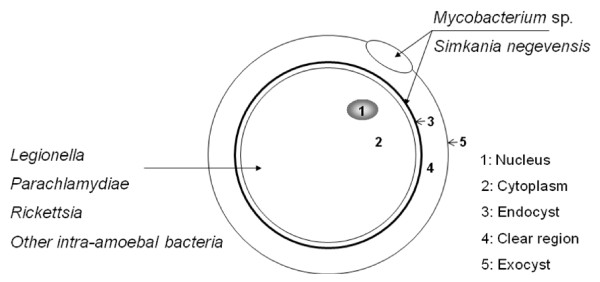
**Preferential localisation of *Mycobacterium *sp. and other amoeba-resistant bacterial organisms in amoebal cyst**.

Electron microscopy further disclosed that the 11 MAC strains under study were entrapped inside of the *A. polyphaga *cysts (Figure 2C, D). In all cases, the intracystic organisms were localized within the exocyst. In addition, *M. marseillense *could be observed in the clear region between the exocyst and the endocyst and in the inner side of the endocyst, and this was also the situation for *M. intracellulare *(Figures 2C, D) (Table 2). We further observed that a 36-hour exposure of the cysts to HCl did not affect the viability of the cysts, as new trophozoites emerged after 7-day incubation in peptone yeast extract-glucose (PYG) media at 32°C as determined by light microscopy. Sub-culturing such trophozoites on Middlebrook 7H10 agar yielded mycobacteria for all of the 8 MAC species (11 strains) under study after a 15-day incubation, whereas the cyst washing fluid remained sterile. Interestingly, we observed that these mycobacteria occupied a preferential location within the amoebal exocyst, where they were found in-between the two layers of the exocyst. Among the several *Mycobacterium *species reported to survive within amoebal cysts, such a particular feature has been previously illustrated only for *M. avium *in *A. polyphaga *cysts [21]; *M. smegmatis *[37]; *M. abscessus*, *M. chelonae *and *M. septicum *[3]; and *M. xenopi *[38]. Among intra-amoebal bacteria, location within the exocyst has also been reported for *Simkania negevensis *[39], despite the fact that *S. negevensis *organisms could also be observed within the cytoplasm of the cyst, depending on the strain under study [40]. Location within exocyst wall contrasts with the observation of *Legionella pneumophila*, which was found within the cytoplasm of pre-cysts and mature cysts of *A. polyphaga *[41] or non-entrapped within amoebal cysts [42]. Reviewing published data regarding amoebal-resistant bacterial species [1,2] found that 11/32 (34.37%) *Mycobacterium *species versus 1/28 (3.57%) non-mycobacterium amoebal-resistant bacterial species have been reported to survive within *A. polyphaga *exocyst (*P *= 0.003) (Figure 3). As both L. pneumophila and mycobacteria are pathogens, the intracystic location of organisms may not influence their virulence. The mechanisms and biological significance of this particular location remain to be studied. It has been established that *A. polyphaga *exocyst is composed of cellulose [43] and the authors have observed that mycobacteria encode one cellulose-binding protein and one or two cellulases which are indeed transcribed [44]. Cellulase encoded by mycobacteria may play a role in their unique exocyst location.

**Table 2:** Abundance of mycobacteria in *A. polyphaga *strain Linc-AP1 and their preferential location in amoebal cyst wall.

MAC species	No. of vacuoles that contain mycobacteria	Location in amoebal cyst wall
*M. timonense*	1.3 ± 0.5 vacuoles	Exocyst
*M. bouchedurhonense*	2.1 ± 1.7 vacuoles	Exocyst
*M. marseillense*	2.4 ± 1.4 vacuoles	Exocyst, clear region, cytoplasm
*M. avium *(*M. avium *subsp. *avium*)	2.6 ± 2.2 vacuoles	Exocyst
*M. chimaera*	3.6 ± 2.6 vacuoles	Exocyst, cytoplasm
*M. intracellulare*	4.6 ± 4.8 vacuoles	Exocyst, Endocyst
*M. colombiense*	5.7 ± 6.2 vacuoles	Exocyst, cytoplasm
*M. arosiense*	9.4 ± 15.2 vacuoles	Exocyst

Moreover, we observed that all MAC species can survive within such *A. polyphaga *cyst. This occurrence did not merely result from the potential contamination of the amoeba by extra-amoebal mycobacteria, since we destroyed any MAC organism left on the surface of cysts by incubating the cysts in HCl, a method previously demonstrated to kill remaining trophozoites, immature cysts and extra-amoebal *M. avium *[21]. We checked the efficacy of this process by incubating the rinsing buffer on Middlebrook and found no growth of mycobacteria, which indicated that the HCl had indeed destroyed any extracystic MAC organisms. The fact that all of the MAC species survived in the exocyst may be relevant to the persistence of these organisms in the environment despite adverse conditions. Non-tuberculous mycobacteria, including *M. avium*, have been shown to persist up to 26 months in drinking water systems despite filtration and ozonation [45]. Also, *M. intracellulare *and other non-tuberculous mycobacteria have been shown to be protected against 15 mg/liter of free-chlorine for 24 hours by entrapment within *A. polyphaga *cysts [3]. Therefore, free-living amoeba cysts may be a "Trojan horse" for MAC organisms and protect them from adverse environmental conditions, including high concentrations of chlorine, as previously reported for other environmental mycobacteria.

## Conclusion

The data presented herein on MAC species illustrate that survival within the amoebal exocyst is a significant feature of environmental mycobacteria. This particular location, preserving mycobacteria from adverse environment, nevertheless allow them to rapidly escape from the amoebal cyst. The mechanisms for such unique location remain to be established in environmental mycobacteria.

## Methods

### Mycobacterium strains

*M. avium *subsp. *avium *ATCC 25291^T^, *M. chimaera *DSM 446232^T^, *M. colombiense *CIP 108962^T^, *M. arosiense *DSM45069^T ^[33], *M. marseillense *CSURP30^T^, *M. timonense *CSURP32^T ^and *M. bouchedurhonense *CSURP34^T ^[35] reference strains that were previously identified by 16S rRNA and *rpo*B gene sequencing [34] were subcultured on Middlebrook 7H10 agar (Becton Dickinson, Le Pont de Claix, France) for 7 days at 30°C under a 5% CO_2 _atmosphere. Cells were washed in 1.5 ml phosphate buffered saline (PBS), pH 7.3, by centrifugation at 8,600 *g*, and the inoculum was adjusted to 10^6 ^bacteria/ml in PBS.

### Infection of amoeba

The *A. polyphaga *strain Linc-AP1 was obtained from T. J. Rowbotham, Public Health Laboratory, Leeds, United Kingdom and cultured at 28°C for 3 days in 150 cm^3 ^culture flasks (Corning, New York USA) that contained 30 ml PYG broth [46]. Amoebal cells were harvested by centrifugation at 500 *g *for 10 min. The pellet was suspended twice in PAS to obtain 5 × 10^5 ^cells/ml. One milliliter of this suspension was dropped into each well of a 12-well microplate (Corning) and incubated at 33°C for 7 days. The microplate, prepared as described above, was used for culturing the mycobacteria. Each well of the microplate was inoculated with a final concentration of 10^6 ^mycobacteria/ml (MOI = 10). The inoculum was sonicated for 5 min at 234 watts (BRANSON 2210; Branson Ultrasonics Corporation, Danbury, CT, USA) in order to limit mycobacteria cell clumping. The microplate was centrifuged at 1,000 *g *for 30 min and incubated at 33°C under a humidified, 5% CO_2 _atmosphere. This microplate was examined daily for 15 days for cytopathic effects and the presence of intra-amoebal organisms by shaking, cytocentrifugation at 200 *g *for 10 min and Ziehl-Neelsen staining.

### Encystment and excystment of infected amoeba

In 25 cm^3 ^culture flasks (Corning), 10 ml of amoeba that had been infected for 48 hours were rinsed once with encystment buffer adapted from [21] (0.1 M KCl, 0.02 M Tris, 8 mM MgSO_4_, 0.4 mM CaCl_2_, 1 mM NaHCO_3_). After centrifugation at 500 *g*, the pellet was resuspended in 10 ml of fresh encystment buffer and incubated for 3 days at 32°C. The excystment of the cysts was examined by light microscopy. Amoebal cysts were pelleted by centrifugation at 1,000 *g *for 10 min and treated with 3% (vol/vol) HCl as previously described [21]. Treated cysts were then washed three times with PAS buffer. Half of the sample was processed for electron microscopy (see above), and the other part was incubated for 7 days in PYG medium at 33°C. Intra-amoebal mycobacteria were released by lysing the monolayer with 1 ml of 0.5% sodium dodecyl sulfate, followed by two successive passages through a 27-gauge needle [3]. The presence of viable mycobacteria was documented by detecting colonies on Middlebrook 7H10 agar inoculated with 200 μl of the cell lysate and incubated at 30°C for 15 days. The identities of the mycobacteria were confirmed by Ziehl-Neelsen staining and partial *rpo*B gene sequencing using primers Myco-F (5'-GGCAAGGTCACCCCGAAGGG-3') and Myco-R (5'-AGCGGCTGCTGGGTGATCATC-3') [34]. All experiments were repeated three times.

### Electron microscopy

Non-ingested mycobacteria were eliminated by rinsing the amoebal monolayer twice with sterile PBS. The amoeba monolayer that was previously infected by MAC species was then fixed in 2% glutaraldehyde and 0.1 M cacodylate buffer overnight. After this first fixation, the bacteria were fixed in 2% glutaraldehyde and 0.33% acroleine in a 0.07 M cacodylate buffer for 1 hour. After washing in 0.2 M cacodylate buffer, the bacteria were post-fixed in 1% osmium bioxide in 0.1 M potassium ferrycyanure for 1 hour and dehydrated in an ascending series of ethanol concentrations, and after 100% ethanol, the dehydration was finished in propylene oxide, and the samples were embedded in an Epon 812 resin. Sections (70 nm) were stained with 5% uranyl acetate and lead citrate before examination with a transmission electron microscope (Philips Morgagni 268D, Eindhoven, the Netherlands). For the determination of mycobacterial abundance, we made observations on a total of 30 *A. polyphaga *trophozoites for each of the 8 MAC species. In order to determine the total number of mycobacteria per trophozoite, we recorded the total number of vacuoles with one *Mycobacterium *organism and the total number of vacuoles with > 1 *Mycobacterium *organism. We also made observations on a total of 30 *A. polyphaga *organisms for each of the 8 MAC species in order to determine their intracystic location, which was considered as intracystic when apposed to the cyst wall and reaching into the cyst wall (between the endo- and the exocyst). These observations were performed in triplicate.

### Statistical tests

Comparison among amoeba-resistant bacterial species [2] as for their survival within exocyst was done using the χ^2 ^test and corrected by Mantel Haenszel method. Comparaisons of mean ± standard deviation of the number of infected vacuoles were done using the ANOVA test. A *P *value < 0.05 was considered to be significant.

## Competing interests

The authors declare that they have no competing interests.

## Authors' contributions

IBS performed the experiments, he interpreted data and wrote the manuscript.

MD designed the experiment, he provided support, interpreted data and wrote the manuscript. Both authors have read and approved the final version of the manuscript.
